# Risk factors and personality characteristics of nonsuicidal self-injurious behavior in clinical sample of female adolescents

**DOI:** 10.1192/j.eurpsy.2024.926

**Published:** 2024-08-27

**Authors:** A. Osváth, C. Hankó, M. Csáki, E. Molnár, J. Pahocsa, S. Kocsor, K. Tóth, D. Fertői, K. A. Sándor-Bajusz, T. Dergez, G. Csábi

**Affiliations:** ^1^Child and Adolescent Psychiatry; ^2^Division of Child and Adolescent Psychiatry, Department of Pediatrics, Medical School and Clinical Center; ^3^Institute of Bioanalysis, Medical School and Clinical Center, University of Pécs, Pécs, Hungary

## Abstract

**Introduction:**

Nonsuicidal self-injury (NSSI) is a self-damaging behavior with typical onset in early adolescence, and shows greater prevalence in females. NSSI is defined by recurrent episodes of intentional self-inflicted damage to body tissue, without suicidal intent. These recurring self-inflicted injuries are done by the indivuduel to relief oneself from negative feelings, to resolve interpersonal difficulties, or to induce positive feelings.

NSSI in DSM-5. has been included among the conditions in need of further study.

NSSI can be interpreted as a maladaptive coping mechanism that can be regarded as an emotional dysregulation. Adverse childhood experiences including physical abuse, neglect or sexual abuse are the strongest predictors of the NSSI. Research has repetitively found strong associations between NSSI and identity diffusion and/or distorted personality traits.

**Objectives:**

The aim of our study was to assess the association between childhood traumatization, personality characteristics including stages of identity development, and self-injurious behavior among female adolescents that experience difficulties with emotional regulation.

**Methods:**

We compared our results to a Hungarian normative sample. The sample consisted of inpatients adolescents, age between 14 and 18, with a diagnosis consisting of „Emotional disorders with onset specific for childhood” or „Mixed disorders of conduct and emotions” with chronic nonsuicidal self-injurious behavior.

Childhood traumatization was measured with the short version of Childhood Trauma Questionnaire (H-CTQ-SF). Identity development and identity diffusion were measured with the Assessment of Identity Development in Adolescence Questionnaire (AIDA). Temperament and character factors were measured with the Junior Temperament and Character Inventory (JTCI).

**Results:**

Adolescent patients with NSSI reported severe and multiplex childhood traumatization. These patients scored higher on novelty seeking and harm avoidance and scored lower on the persistence, self-directedness, and cooperativeness factors in the JTCI. These results were compared with the Hungarien normative sample. Adolescents scored higher on the Discontinuity and Incoherence scales of the AIDA.

**Conclusions:**

Our preliminary results suggest that childhood traumatization predicts self-injurious behavior in adolescent females. Additionally, we have found associations between signs indicative of personality disorder, including lower level of self-cohesion, self-integration and self-directedness.

**Disclosure of Interest:**

None Declared

